# Capacitive spectroscopy as transduction mechanism for wearable biosensors: opportunities and challenges

**DOI:** 10.1007/s00216-023-05066-y

**Published:** 2023-12-13

**Authors:** Ana Díaz-Fernández, Noemí de-los-Santos-Álvarez, María Jesús Lobo-Castañón

**Affiliations:** 1 Departamento de Química Física y Analítica, Universidad de Oviedo, Av. Julián Clavería 8, 33006 Oviedo, Spain; 2 Instituto de Investigación Sanitaria del Principado de Asturias, Avenida de Roma, 33011 Oviedo, Spain

**Keywords:** Capacitance, Chemical sensors, Non-invasive monitoring, Wearable sensors

## Abstract

Wearable sensors would revolutionize healthcare and personalized medicine by providing individuals with continuous and real-time data about their bodies and environments. Their integration into everyday life has the potential to enhance well-being, improve healthcare outcomes, and offer new opportunities for research. Capacitive sensors technology has great potential to enrich wearable devices, extending their use to more accurate physiological indicators. On the basis of capacitive sensors developed so far to monitor physical parameters, and taking into account the advances in capacitive biosensors, this work discusses the benefits of this type of transduction to design wearables for the monitoring of biomolecules. Moreover, it provides insights into the challenges that must be overcome to take advantage of capacitive transduction in wearable sensors for health.

**Graphical abstract:**

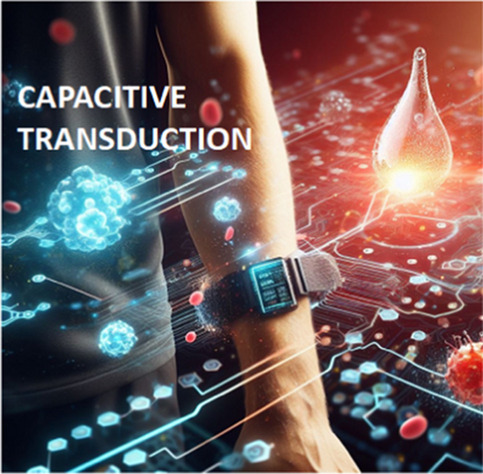

## Introduction

Wearable sensors are devices designed to be worn on or attached to the body, typically as accessories or clothing, to collect data about various physiological, environmental, or contextual parameters. They empower individuals to track and manage their health and lifestyle and, as such, offer great potential to move towards personalized medicine. Despite wearables have gained significant popularity in recent years due to advances in miniaturization, wireless communication, and sensor technology, most commercially available wearable devices are sensors for monitoring physical parameters such as heart rate, temperature or body motion [1]. However, to achieve a comprehensive health monitoring, it is necessary to obtain more detailed information at the molecular level, that is, to be able to continuously and real-time monitor the concentration of specific molecules in easily accessible biofluids such as saliva, sweat, tears or interstitial fluid. This is accomplished by integrating a molecular recognition element, such as enzymes, antibodies, aptamers or cell receptors into the wearable operation thus obtaining wearable biosensors [2–4].

An example of a successful wearable biosensor is the transdermal glucose monitoring device, which specifically measures the continuous status of diabetes relying on the advances in enzyme electrodes over the last decades. However, this impressive development is not generalizable and new sensing mechanisms should be explored to expand the range of applications of wearable sensors for human health monitoring [4]. This involves not only developing new biomimetic recognition elements such as aptamers or molecularly imprinted polymers to specifically recognize the target analytes, but also testing novel transduction methods that convert the chemical information into a measurable signal, in a reagent-less, fast and continuous operation.

In recent years, capacitive transduction has emerged as an attractive alternative for biosensors development because of their simplicity, low cost and low power consumption, fast response time and high sensitivity [5–7]. This trends article summarizes the basic functioning mechanism of capacitive spectroscopy sensors, placing particular attention on their benefits for the development of wearable biosensors. We then present what we view as the main challenges and opportunities to create effective and reliable capacitance-based wearable biosensors.

## Mechanisms of capacitive spectroscopy chemical sensors

Capacitance spectroscopy, which measures changes in capacitance as a function of frequency, is a suitable transduction mechanism for developing wearable biosensors. By applying a potential to a blocked sensing interface, it acts as a capacitor, and its capacitance given by $C=\varepsilon {\varepsilon }_{0}\frac{A}{d}$, where ε is the dielectric constant of the medium, ε_0_ the vacuum permittivity, A is the surface area and d the thickness of the dielectric layer, is a measure of its ability to store electric charge [6]. The specific interaction of the analyte with the sensing phase alters its dielectric properties leading to changes in capacitance, which can be accurately related with the analyte concentration.

**Fig. 1:**
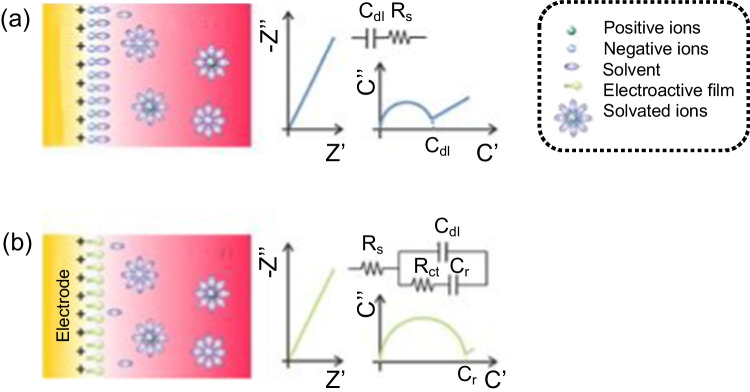
Schematic illustration of reagent-less non-faradaic (**a**) and faradaic (**b**) modes used in capacitive sensors. On the right are shown the characteristic spectra in the impedance and capacitive planes and the equivalent circuits to model the interface. Adapted from Ref. [11] with permission form American Chemical Society, copyright 2019

In impedance-based capacitance spectroscopy, an alternating excitation is used, imposing a sinusoidal voltage on the sensing phase whose frequency is swept over a wide frequency range (typically 1 MHz to 0.01 Hz), and measuring the resulting current. Impedance (Z*) is acquired as the ratio between the applied voltage and the current, both frequency-dependent, and then converted to capacitive data (C* = 1/jωZ*). There are two different modes of measurement, non-faradaic (in the absence of electroactive probes) and faradaic (in the presence of a redox probe), with the possibility of using different types of electrochemical interfaces (Fig. 1). Both are non-destructive methods, sensitive to changes in sensor interface without requiring for added reagents, and thus suitable for wearable devices.

The simplest interface is formed when the sensing phase, constructed on a conductive surface without any electroactive probe, is brought into contact with an electrolyte solution, achieving the subsequent charge equilibrium (Fig. 1a). This interface is modelled by a capacitance in series with a resistance, which corresponds to the capacitance of the double layer (C_dl_) and the resistance of the solution (R_s_). The molecular recognition at the interface alters C_dl_, which is used as the signal. Typically, the capacitance response is monitored at a single frequency, selected as the frequency at which the maximum capacitance change occurs upon analyte binding.

The most common faradaic mode, not shown in Fig. 1, involves the use of redox probe in solution, using the associated charge transfer resistance (R_ct_) as sensing parameter. The need to add a reagent to the sample invalidates this mode for the design of wearables. However, there is another type of less known faradaic mode, which is suitable for reagent-less assays (Fig. 1b). It involves the use of a redox probe linked to the sensing phase, either as an additional component to the specific receptor [8] or as a label of the receptor itself [9]. The inclusion of a redox probe in the interface provides new elements to the equivalent circuit, which are the electron transfer resistance (R_ct_) and a redox capacitance (C_r_). The latter is a specific type of capacitance strictly associated with a redox reaction, which takes into account the charge of the redox probes and how they communicate with electrode states [10].

## Benefits of capacitive sensors for wearable devices

Capacitive spectroscopy-based sensors provide valuable information to be used in the detection of biomarkers of great interest, in a reagent-less approach directly in the living body. We describe here some examples where capacitive spectroscopy biosensors have been successfully applied, although not in a wearable format. Additionally, the construction and performance of wearable sensors with this transduction principle is described, which can serve as inspiration to expand the number of these devices with improved assay capabilities.

Capacitive biosensors have been used for the detection of a wide range of analytes (Table 1). This transduction approach is generalizable and compatible with the use of different molecular recognition elements: immunoreagents, both antigens for the recognition of antibodies [12, 13] and antibodies for the recognition of proteins [14] or whole cells [15]; synthetic oligonucleotides for detecting RNA targets on the basis of the hybridization reaction [16] and aptamers for monitoring the corresponding targets after the affinity interaction [17, 18]. Synthetic peptides or affimers [19] and macrocyclic compounds such as β-cyclodextrins [20] have also been employed in the design of the selective layer. The molecular recognition interaction in the sensing phase causes a change in the charge distribution, surface dielectric properties or local conductance, which is correlated with the analyte concentration. This sensing mechanism is capable of detecting the analytes without user intervention, making the strategy suitable for point-of-care diagnostics.

**Table 1:** Non-wearable reagentless capacitive sensors, non-faradaic (NF) and faradaic (F)

Analyte	Sensing design	Media	Working range / LOD	Selectivity	Ref
COVID-19 Ab	Trimeric spike protein/EDC-NHS/-NH_2_/Au-glass; NF	Serum	1 – 100 BAU mL^−1^ 0.4 BAU mL^−1^	Negative serum	[12]
anti-rT24H Ab	Antigen/Au electrode; NF	Serum	0.1 – 100 pg mL^−1^ 24.1 fg mL^−1^	BSA	[13]
SARS-CoV2-nucleoprotein	Ab/PEDOT:PSS vertically electrode; NF	viral fluids	4.1 ng mL^−1^	–	[14]
CD34 ^+^ -T-Cells	Anti CD34^+^-Ab/EDC-NHS/MUA + MCH/Au electrode; NF	2% serum	50 – 105 cell mL^−1^ 44 cell mL^−1^	HL-60 and C2C12 cells	[15]
mRNA	ssDNA-Au-PCB; NF	Serum	0.1 fM – 1 pM 323 aM	Negative serum	[16]
Amyloid beta	aptamer/APTS SAM/Au electrode; NF	Plasma	1 fM – 1 nM 0.1 fg mL^−1^	BSA, glutaraldehyde	[17]
IL-6	Aptamer + MCH SAM/Au electrode; NF	10% serum	10 pg mL^−1^	MMP3 protein	[18]
HER4	Affimer/Au interdigitated electrode; NF	Serum	1 pM – 100 nM 1 pM	PSA, thrombin	[19]
Cortisol	PPG:βCD/glassy carbon; NF	Urine	1.29 nM	uric acid, acetaminophen, resveratrol	[20]
		Saliva	1.33 nM		
NS1 Flavivirus	aptamer/redox-peptide support/Au electrodes; F	serum	5 – 1000 ng mL^−1^ 0.5 ng mL^−1^	Fetuin	[21]
C-reactive protein	Aptamer/peptide support/ Au electrode; F	PBS buffer	10 – 5000 pM	BSA	[22]
IL-6	Antibody/PBNP/GO/GCE; F	buffer	5.6	Fetuin	[23]
β-1,4-GalT-V	Ab/AuNRs-Prussian blue/ SPCEs; F	Serum	50—400 fM 20 fM	p53, anti-p53 Ab, IL-8 cytokine, IgG Ab	[24]

*Ab* antibody, *APTS* (3-aminopropyl)triethoxysilane, *AuNRs* gold nanorods, *βCD* beta-cyclodextrin, *BSA* bovine serum albumin, *EDC* 1-ethyl-3-(3-dimethylaminopropyl)carbodiimide, *GCE* glassy carbon electrode, *GO* graphene oxide, *HER4* human epidermal growth factor receptor 4, *MCH* 6-mercaptohexanol, *MMP-3* matrix metalloproteinase-3, *mRNA* microRNA, *MUA* 11-mercaptoundecanoic acid, *NHS* N-hydroxysuccinimide, *PBNP* prussian blue nanoparticles, *PCB* printed circuit board, *PEDOT* poly(3,4-ethylenedioxythiophene), *PPG* polypropylene glycol, *PSA* prostate specific antigen, *PSS* poly(styrenesulfonate), *rT24H* recombinant *Taenia solium* antigen, *SAM* self-assembled monolayer, *ssDNA* single stranded DNA, *β-1,4-GalT-V* β-1,4-galactosyltransferase-V

Interdigitated electrodes are the typical substrates for construction, which are easy to miniaturize, although the insulating/immobilization chemistries are critical to ensure stable and reproducible measurements. The use of an AC excitation signal can simultaneously accelerate the electrokinetic convection of the analytes to the sensing phase, thus shortening the analysis time and improving the response. For example, *Jiang *et al*.* developed a sensor for the detection of microRNA (mRNA) extracted from vesicles directly in bovine serum in as little as 30 s with a detection limit (LOD) of 323 aM and a linear dynamic range from 0.1 fM to 1 pM in diluted serum. The sensor has been successfully used in the analysis of clinical cow samples [16]. Larger molecules can also be detected with a greater change in capacitance, resulting in more sensitive biosensors. This is the case of the sensor that detects COVID-19 antibodies in serum using an interdigitated microelectrode array modified with the trimeric spike protein of COVID-19 by the activation of -NH_2_ groups on the surface. The sensor has a LOD of 0.4 binding antibody units (BAU) mL^−1^ and a working range of 1 to 100 BAU mL^−1^, allowing the analysis of samples in only 1 h [12].

The sensitivity of the devices is also improved by using multiple vertically paired electrodes made with a conducting polymer, PEDOT:PSS, as the electrode material and a parylene film dielectric layer, which enabled a very short and reproducible electrode gap. This led to a LOD that met the requirements for medical diagnosis of COVID-19 [14].

In a step towards adapting the capacitive biosensor to a portable and wearable device, these platforms can be integrated into a microfluidic system for continuous monitoring the analyte. This strategy has been demonstrated for the detection of CD34^+^-T-cells in 2% serum, using gold electrodes modified with a monolayer of mercaptoundecanoic acid and mercaptohexanol for anchoring the specific anti-CD34^+^ antibody. The sensor has a LOD of 44 cell mL^−1^, a working range between 50 and 10^5^ cell mL^−1^ and excellent selectivity to other cell types [15]. In addition, it is possible to construct the sensor on a flexible substrate more suitable for wearable devices using a thin-film gold sensor assembly with multiple channels. For example, for the detection of interleukin-6 (IL-6) in 10% human serum, the gold surface has been modified with a specific IL-6 aptamer. The sensor has a LOD of 10 pg mL^−1^, which is within the pathological range for the diagnosis of inflammatory diseases [18].

Non-faradaic capacitive sensors support measurements not only in serum or plasma samples but also in extracellular fluids such as saliva or sweat. For instance, *Panahi *et al*.* detected cortisol in saliva using a polypropylene glycol: cyclodextrin glassy carbon with a LOD of 1.3 nM and great selectivity towards various compounds such as uric acid, acetaminophen and resveratrol. Also, the sensor is reusable with a lifetime of 10 cycles and a stability of 1 month [20].

Additionally, reagent-less faradic mode capacitive spectroscopy could represent a great approach for developing wearable devices, combining the advantages of faradic mode impedance with the reagent-less mode of capacitive spectroscopy. In reagent-less faradaic mode, it is essential that the tethered redox probe is confined to a 10 nm thickness layer. Its distance to the electrode surface is usually fixed by either a covalent link to an irrelevant scaffold (e.g. a redox-tagged alkanethiol [21] or peptide [22]) or direct deposition on the surface (e.g. Prussian Blue nanostructures) [23, 24]. The second strategy yields improved sensitivity (fM vs pM) and stability of the layer in aqueous environment, but requires an additional layer to immobilize the receptor, typically an antibody, on graphene [23] or Au nanostructures [24]. The redox-tagged peptide is especially well-suited for small receptors such as aptamers to increase the capture efficiency because they are covalently linked to the peptide. The higher receptor surface density can significantly improve the LOD by more than 30 times [22]. Both strategies were successfully tested in biological fluids, even in undiluted serum with minimal loss in sensitivity [24].

The redox probe can also be linked to the receptor, and its distance to the surface is modulated by the ligand interaction. In such cases, the most sensitive circuit element is not the redox pseudocapacitance but the electron-transfer resistance [9]. There is also a shift in the phase angle. Though the magnitude is small it might be used for measurement because it would support extraordinarily rapid signal acquisition, fundamental in wearable devices. This approach is relatively insensitive to unspecific adsorption and oxygen reduction.

**Fig. 2:**
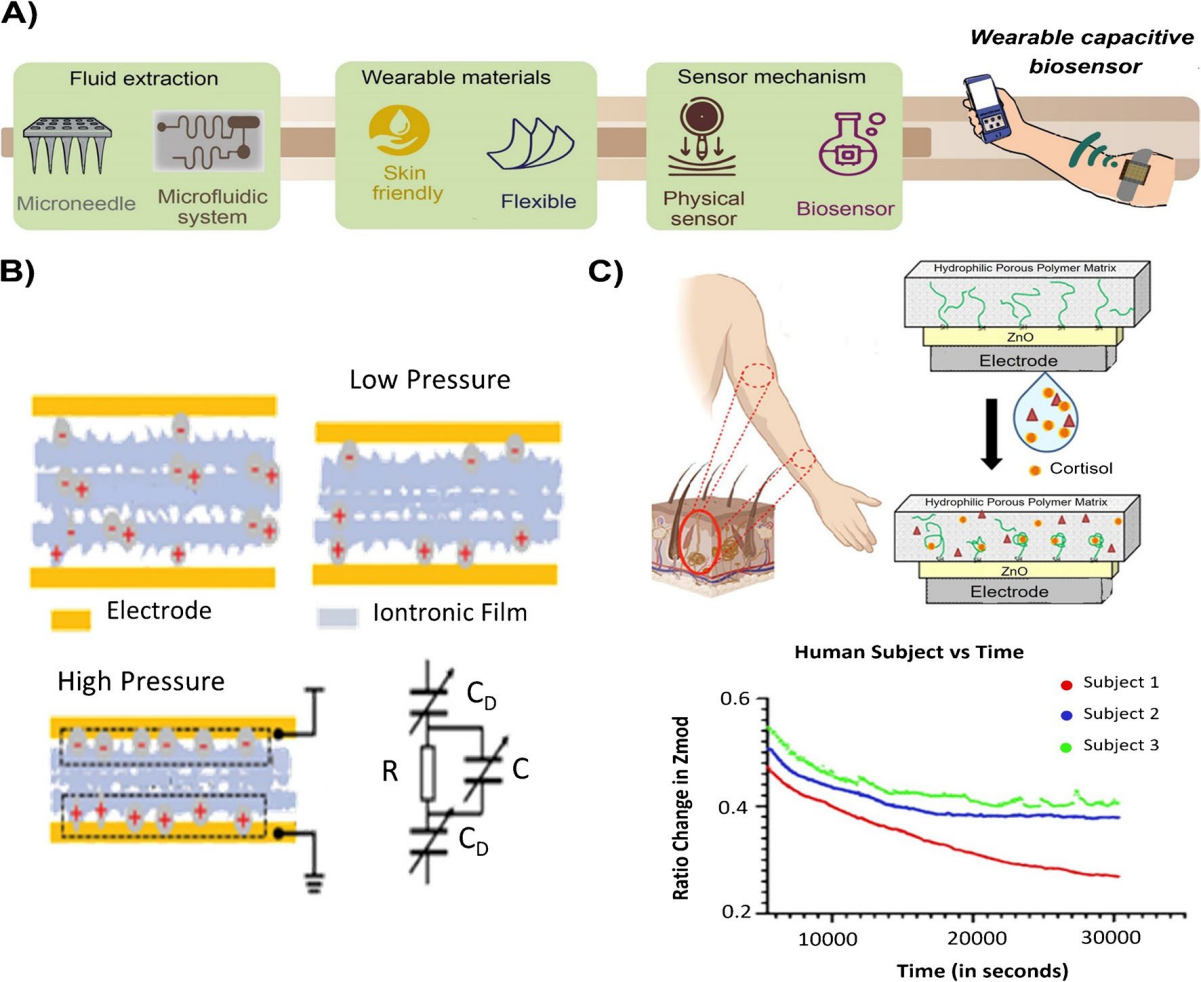
Towards wearable capacitive biosensors. **A**) Main components of a wearable device. **B**) Wearable physical sensor (adapted from ref. [25]). **C**) Wearable capacitive biosensor (adapted from ref. [29])

The capacitive sensors discussed so far are characterized by a simple structure and easy operation, with excellent analytical characteristics that match the analytical performance for point-of-care medical devices. However, for a wearable format they must also meet other key requirements such as flexibility and biocompatibility, stretchability, real-time monitoring, stability and be comfortable to wear for extended periods. Capacitive wearable sensors have been described for collecting physiological parameters such as blood pressure, body temperature and humidity (Fig. 2B) [25]. This type of sensors might be integrated in wearable biosensors to minimize the contribution of these parameters on the performance. However, a challenge in wearable devices is minimizing the noise signal induced by human motion. Flexible biosensors with conformability like that of skin would alleviate it [26].

Despite the increasing number of works reporting on wearable chemical sensors, only four of them have used capacitive spectroscopy as a readout technique. *Wei *et al. reported an epidermal microfluidic patch sensor for simultaneous monitoring of sweat rate and chloride concentration using the admittance signal [27]. *Song *et al*.* designed an immunosensor for the quantification of human interleukin 8 (hIL8) in mouse blood. They used a microfabricated needle-shaped microwell sensor that allows transcutaneous or intramuscular insertion. The sensor supports real-time readout and specificity (no response in control mice) ant the potential of multiplexing. However, some issues still need further development. The linear correlation is weak in the whole wide dynamic range claimed and the microneedle design should be optimized to avoid damage [28].

Real-time tracking and monitoring are enabled in many wearable sensors that are equipped with wireless connectivity (e.g., Bluetooth, Wi-Fi) to transmit data to smartphones, tablets, or computers for further analysis and display. To achieve this, the performance during long periods and the saturation effects should be considered. However, this is not enough, autonomous sensing of the device, that is, passive extraction of the biofluid, is also mandatory. *Ganguly *et al*.* reported a wristband aptasensor for continuous and autonomous monitoring of cortisol over 8 h without saturation (Fig. 2C). They used a nanoporous ZnO silver electrode modified with the specific aptamer. The sensor substrate consists of a polyamide membrane that is biocompatible and skin-friendly and permits percolation of the unstimulated excreted sweat in less than 1 min. This sensor design allows dynamic detection of cortisol from 1 to 256 ng mL^−1^, from low to high concentration and vice versa, supporting calibration for daily variations. Furthermore, the sensor shows no cross-reactivity with other substances [29]. In a step forward, *Churcher* et al. developed a platform for the simultaneous detection of cortisol and NPY in sweat. The platform (AptaStrentror) integrates the aptasensor with a portable potentiostat in a 3D printed housing, which enables the placement on the wrist [30].

## Outlook

While there are numerous opportunities in the development of capacitance-based wearable biosensors, there are also significant challenges that researchers and engineers must address to create effective and reliable devices. Some of the key challenges include:

### Materials and biocompatibility

Ensuring that the materials used in the construction of the sensors are not only flexible and stretchable but also biocompatible, non-toxic, and safe for body contact is essential when developing wearable sensors [31]. Allergic reactions or skin irritation must be prevented. In addition, wearable sensors should be comfortable to wear for extended periods. Achieving a balance between sensor size and user comfort is essential. Designing devices that are aesthetically pleasing, easy to use, and integrate seamlessly into daily life is important for user adoption.

### Miniaturization

Shrinking capacitive sensors without sacrificing performance is another technical challenge. Modern bioelectronic fabrication technology has enabled multianalyte detection in real-time. When compared with omics technologies that detect hundreds of genes or metabolites at a time, there is still room for improvement. The number of integrated biosensors in a wearable device is defined by the physical space needed to achieve reliable and robust measurement on biocompatible materials in a harsh environment that promotes biofouling. Most wearable sensors rely on batteries for power, and it is important to optimize power consumption to extend battery life during continuous monitoring while maintaining sensor performance. It is also crucial to secure wireless data transmission between the wearable sensor and external devices such as smartphones or cloud servers to offer the end users with valuable and timely chemical information.

### Sampling methods

The biofluids most commonly sampled are sweat and interstitial fluid (ISF). Advanced methods for reliable and reproducible collection of these samples in a continuous way are required. The simplest way to collect sweat is by capillarity towards the pores of polymeric membranes directly in contact with the skin. The main drawback of this method is the small amount of sample obtained. Alternatively, the sample can be extracted using iontophoresis [31]. Microneedles and iontophoretic extraction are the approaches used for obtaining ISF, although it would be necessary to develop improved systems that allow rapid extraction with reduced irritation for continuous and prolonged use of these devices.

### Calibration and drift

The sensor components must remain stable over time. Sensor drift is a common problem that affects the reliability of measurement, and correcting this drift is crucial. The development of antifouling materials, as well as robust calibration methods such as dual sensor configuration for relative measurements are required to assure long-term stability and accuracy.

### Multiplexed analysis

To achieve a more accurate prediction of a patient's physiological state, it will be necessary to develop different sensors on the same platform to simultaneously monitor a broader range of biomarkers. Moreover, the complexity of daily tracking, circadian fluctuation of biomarker concentration and variable operational conditions (e.g. changes in skin temperature) would impose an additional desirable feature: real time recalibration upon simultaneous readings of other physical and/or chemical parameters that impact the accuracy of the main analytes [32].

Meeting these challenges will require multidisciplinary collaboration among scientists, engineers, healthcare professionals, and user experience designers. Additionally, ongoing research and innovation in materials science, sensor technology and data analysis will be essential to advance the field of capacitive wearable biosensors.

## References

[1] Bandodkar AJ, Jeerapan I, Wang J (2016). Wearable chemical sensors: present challenges and future prospects. ACS Sens.

[2] Kim J, Campbell AS, Esteban-Fernádez de Ávila B, Wang J (2019). Wearable biosensors for healthcare monitoring. Nat Biotechnol.

[3] Yang Y, Gao W (2019). Wearable and flexible electronics for continuous molecular monitoring. Chem Soc Rev.

[4] Flynn CD, Chang D, Mahmud A, Yousefi H, Das J, Riordan KT, Sargent EH, Kelley SO (2023). Biomolecular sensors for advanced physiological monitoring. Nat Rev Bioeng.

[5] Huang L, Zhang C, Ye R, Yan B, Zhou X, Xu W (2024). Capacitive biosensors for label-free and ultrasensitive detection of biomarkers. Talanta.

[6] Kirchhain A, Bonini A, Vivaldi F, Poma N, Di Francesco F (2020). Latest developments in non-faradaic impedimetric biosensors: towards clinical applications. Trends Anal Chem.

[7] Weaver S, Mohammadi MH, Nakatsuka N (2023). Aptamer-functionalized capacitive biosensors. Biosens Bioelectron.

[8] Santos A, Piccoli JP, Santos-Filho NA, Cilli EM, Bueno PR (2015). Redox-tagged peptide for capacitive diagnostic assays. Biosens Bioelectron.

[9] Downs AM, Gerson J, Ploense K, Plaxco KW, Dauphin-Ducharme P (2020). Sub-second-resolved molecular measurements using electrochemical phase interrogation of aptamer-based sensors. Anal Chem.

[10] Bueno PR, Fernandes FCB, Davis JJ (2017). Quantum capacitance as a reagentless molecular sensing element. Nanoscale.

[11] Garrote BL, Santos A, Bueno PR (2019). Perspectives and precautions for the uses of electric spectroscopic methods in label-free biosensing applications. ACS Sens.

[12] Shoute LCT, Abdelrasoul GN, Ma Y, Duarte PA, Edwards C, Zhuo R, Zeng J, Feng Y, Charlton CL, Kanji JN, Babiuk S, Chem J (2023). Label-free impedimetric immunosensor for point-of-care detection of COVID-19 antibodies. Microsyst Nanoeng.

[13] Lin X, Jiang Y, Wu JJ, Eda S, Wan N (2022). An alternating current electrokinetics biosensor for rapid on-site serological screening of *Taenia solium* cysticercosis infection. Microchim Acta.

[14] Park JH, Lee GY, Song Z, Bong JH, Chang YW, Cho S, Kang MJ, Pyun JC (2022). Capacitive biosensor based on vertically paired electrodes for the detection of SARS-CoV-2. Biosens Bioelectron.

[15] Díaz-Fernández A, Bernalte E, Fernández-Ramos C, Moise S, Estrela P, Di Lorenzo M (2022). An impedimetric immunosensor for the selective detection of CD34+T-cells in human serum. Sens Actuators B Chem.

[16] Jiang Y, Huang J, Wu J, Eda S (2022). A rapid, sensitive, and simple-to-use biosensor for on-site detection of attomolar level microRNA biomarkers from serum extracellular vesicles. Sens Actuators B Chem.

[17] Sharma PK, Kim E-S, Mishra S, Ganbold E, Seong R-S, Kim YM, Jahng G-H, Rhee HY, Han H-S, Kim DH, Kim ST, Kim NY (2022). Ultrasensitive probeless capacitive biosensor for amyloid beta (Aβ1-42) detection in human plasma using interdigitated electrodes. Biosens Bioelectron.

[18] Sánchez-Salcedo R, Miranda-Castro R, de-los-Santos-Álvarez N, Lobo-Castañón MJ, Corrigan D (2023). Comparing nanobody and aptamer-based capacitive sensing for detection of Interleucin-6 (IL-6) at physiologically relevant levels. Anal Bioanal Chem.

[19] Zhurauski P, Arya SK, Jolly P, Tiede C, Tomlinson DC, Ferrigno PK, Estrela P (2018). Sensitive and selective Affimer-functionalised interdigitated electrode-based capacitive biosensor for Her4 protein tumour biomarker detection. Biosens Bioelectron.

[20] Panahi Z, Ren T, Halpern JM (2022). Nanostructured cyclodextrin-mediated surface for capacitive determination of cortisol in multiple biofluids. ACS Appl Mater Interfaces.

[21] Cechetto J, Fernandes FCB, Lopes R, Bueno PR (2017). The capacitive sensing of NS1 flavivirus biomarker. Biosens Bioelectron.

[22] Piccoli J, Hein R, El-Sagheer AH, Brown T, Cilli EM, Bueno PR, Davis JJ (2018). Redox capacitive assaying of C-Reactive protein at a peptide supported aptamer interface. Anal Chem.

[23] Oliveira RMB, Fernandes FCB, Bueno PR (2019). Pseudocapacitance phenomena and applications in biosensing devices. Electrochim Acta.

[24] Echeverri D, Cruz-Pacheco AF, Orozco J (2023). Capacitive nanobiosensing of β-1,4-galactosyltransferase-V colorectal cancer biomarker. Sens Actuators B.

[25] Cheng AJ, Wu L, Sha Z, Chang W, Chu D, Wang CH, Peng S (2023). Recent advances of capacitive sensors: materials, microstructure designs, applications and opportunities. Adv Mater Technol.

[26] Takaloo S, Zand MM (2021). Wearable electrochemical flexible biosensors: With the focus on affinity biosensors. Sens Biosens Res.

[27] Wei L, Lv Z, He Y, Cheng L, Qiu Y, Huang X, Ding C, Wu H, Liu A (2023). In-situ admittance sensing of sweat rate and chloride level in sweat using wearable skin-interfaced microfluidic patch. Sens Actuators B.

[28] Song N, Xie P, Shen W, Oh H, Zhang Y, Vitale F, Javanmard M, Allen MG (2021). A microwell-based impedance sensor on an insertable microneedle for real-time in vivo cytokine detection. Microsyst Nanoeng.

[29] Ganguly A, Lin KC, Muthukumar S, Prasad S (2021). Autonomous, real-time monitoring electrochemical aptasensor for circadian tracking of cortisol hormone in sub-microliter volumes of passively eluted human sweat. ACS Sens.

[30] Churcher NKM, Greyling C, Upasham S, Lin K-C, Rice P, Pali M, Spiro J, Prasad S (2022). AptaStrensor (aptamer-based sensor for stress monitoring): The interrelationship between NPY and cortisol towards chronic disease monitoring. Biosens Bioelectrons:X.

[31] Ates HC, Nguyen PQ, Gonzalez-Macia L, Morales-Narváez E, Güder F, Collins JJ, Dincer C (2022). End-to-end design of wearable sensors. Nat Rev Mater.

[32] Sempionatto JR, Lasalde-Ramírez JA, Mahato K, Wang J, Gao W (2022). Wearable chemical sensors for biomarker discovery in the omics era. Nat Rev Chem.

